# Evaluation of silk sericin as a biomaterial: in vitro growth of human corneal limbal epithelial cells on Bombyx mori sericin membranes

**DOI:** 10.1186/2194-0517-2-14

**Published:** 2013-11-28

**Authors:** Traian V Chirila, Shuko Suzuki, Laura J Bray, Nigel L Barnett, Damien G Harkin

**Affiliations:** 1 Queensland Eye Institute, South Brisbane, Queensland 4101 Australia; 2 Faculty of Science and Engineering, Queensland University of Technology, Brisbane, Queensland 4001 Australia; 3 Faculty of Health Sciences, The University of Queensland, Herston, Queensland 4029 Australia; 4 Australian Institute of Bioengineering & Nanotechnology, The University of Queensland, St Lucia, Queensland 4072 Australia; 5 Faculty of Science, The University of Western Australia, Crawley, Western Australia 6009 Australia; 6 Faculty of Health, Queensland University of Technology, Brisbane, Queensland 4001 Australia; 7 Max Bergmann Center of Biomaterials, Leibniz Institute for Polymer Research, Dresden, Saxony 01069 Germany; 8 UQ Centre for Clinical Research, The University of Queensland, Herston, Queensland 4029 Australia; 9 Institute of Health and Biomedical Innovation, Kelvin Grove, Queensland 4059 Australia

**Keywords:** Silk, Silk proteins, Sericin, Corneal limbal epithelial cells, Cell attachment

## Abstract

Sericin and fibroin are the two major proteins in the silk fibre produced by the domesticated silkworm, *Bombyx mori*. Fibroin has been extensively investigated as a biomaterial. We have previously shown that fibroin can function successfully as a substratum for growing cells of the eye. Sericin has been so far neglected as a biomaterial because of suspected allergenic activity. However, this misconception has now been dispelled, and sericin’s biocompatibility is currently indisputable. Aiming at promoting sericin as a possible substratum for the growth of corneal cells in order to make tissue-engineered constructs for the restoration of the ocular surface, in this study we investigated the attachment and growth *in vitro* of human corneal limbal epithelial cells (HLECs) on sericin-based membranes. Sericin was isolated and regenerated from the silkworm cocoons by an aqueous procedure, manufactured into membranes, and characterized (mechanical properties, structural analysis, contact angles). Primary cell cultures from two donors were established in serum-supplemented media in the presence of murine feeder cells. Membranes made of sericin and fibroin-sericin blends were assessed *in vitro* as substrata for HLECs in a serum-free medium, in a cell attachment assay and in a 3-day cell growth experiment. While the mechanical characteristics of sericin were found to be inferior to those of fibroin, its ability to enhance the attachment of HLECs was significantly superior to fibroin, as revealed by the PicoGreen^®^ assay. Evidence was also obtained that cells can grow and differentiate on these substrata.

**Electronic supplementary material:**

The online version of this article (doi:10.1186/2194-0517-2-14) contains supplementary material, which is available to authorized users.

## Introduction

Silks are natural polypeptide composite materials commonly included in the group of fibrous proteins. They are produced by certain species, most notably by spiders (order Araneae) and by silkmoths (order Lepidoptera). The silk produced by the larvae of the domesticated silkmoth (*Bombyx mori*) had an important role in the textile industry for millennia, and is, by far, the most extensively studied silk.

The two constitutive proteinaceous molecular complexes in the silks produced by insects are fibroin and sericin. Following seminal studies by Minoura’s group (Minoura et al. 1990,1995a, b), the silk proteins, especially the fibroin produced by the *B. mori* silkworm, have been widely investigated as potential biomaterials and considered for tissue engineering applications (Altman et al. 2003; Wang et al. 2006; Vepari and Kaplan 2007; Hakimi et al. 2007; Kearns et al. 2008; Kundu et al. 2008; Wang et al. 2009; Murphy and Kaplan 2009; Hardy and Scheibel 2010; Numata and Kaplan 2010; Ghassemifar et al. 2010; Harkin et al. 2011; Pritchard and Kaplan 2011; Wenk et al. 2011; Gil et al. 2013).

The assembly of polypeptides known as 'sericin’ represents about one quarter of the total protein content of *B. mori* cocoons. Being soluble in hot water or alkaline aqueous solutions, sericin can be easily removed in a process known as 'degumming’ and isolated as a pure product if needed.

Sericin has been traditionally associated with the immune responses attributed to silk in general (Altman et al. 2003). Sensitization to silk leads to allergic reactions affecting the skin or respiratory tract, while silk as such can also cause an inflammatory response when in direct contact with living tissues. As a result, due to biocompatibility concerns, sericin has been largely neglected as a potential biomaterial. Although still underutilized, there has been a change over the last decade in the attitude regarding the potential applications of sericin in various fields of human activity. This was mainly prompted by environmental and economical concerns with respect to the large amounts of sericin, estimated to exceed 50,000 t annually (Mondal et al. 2007), which have to be discarded as waste from silk processing factories, and also by the realization that sericin is a biocompatible material despite being traditionally regarded as a pathologic allergen. A comprehensive review (Kundu et al. 2008) presents a detailed account of current uses of sericin, chiefly from *B. mori* silkworms, in cosmetics, pharmaceuticals, dietary foods, controlled drug administration, wound dressing, and media for cell culture. Some time ago, it was suggested (Minoura et al. 1995b) that sericin on its own could be a biomaterial, which is obviously different from using it as a supplement in the cell culture medium. However, there have been very few reports on this issue.

We are currently developing and evaluating silk protein substrata or scaffolds for the tissue engineering of the eye (Harkin et al. 2011; Harkin and Chirila 2012), having been the first to assess *B. mori* silk fibroin (henceforward BMSF) as a potential substratum for the restoration of the ocular surface (Chirila et al. 2007,2008,2010). We have further evaluated BMSF membranes as substrata for corneal epithelial constructs (Bray et al. 2011,2012,2013; George et al. 2013), for corneal endothelial constructs (Madden et al. 2011), and for transplantation of retinal cells (Kwan et al. 2010; Shadforth et al. 2012). The potential advantages of BMSF over other materials (e.g. collagen) in tissue engineering applications have been discussed elsewhere (Harkin and Chirila 2012).

In the present report, we investigated the capacity of sericin regenerated from *B. mori* cocoons to function as a substratum for cell growth, either as such or blended with regenerated fibroin. In particular, human corneal limbal epithelial cells (HLECs) were assessed for the first time on sericin substrata with an aim of furthering the use of sericin in tissue-engineered constructs for ocular surface restoration.

## Methods

### Materials

*B. mori* silkworm cocoons were supplied by Tajima Shoji Co Ltd (Yokohama, Japan), with the pupae removed. All chemical reagents were supplied by Sigma-Aldrich (St Louis, MA, USA) with the exceptions mentioned here. Genipin (purity 98%) was purchased from Erica Co Ltd (Xi’an, Shaanxi, China). Water of high purity (Milli-Q or equivalent, Millipore, Billerica, MA, USA) was used in all experiments. Sartorius Stedim Biotech (Göttingen, Germany) supplied the Minisart^®^-GF pre-filters (0.7 μm) and Minisart^®^ filters (0.2 μm). The dialysis cassettes Slide-A-Lyzer^®^ (MWCO 3.5 kDa) were supplied by Thermo Scientific (Rockford, IL, USA) and dialysis tubes with MWCO 12.4 kDa by Sigma-Aldrich. The olefin copolymer Topas^®^ 8007S-04 was purchased from Advanced Polymers (Frankfurt, Germany).

All cell culture reagents and supplements were purchased from Life Technologies (Mulgrave, Victoria, Australia) with the following exceptions: foetal bovine serum (FBS) from Thermo Scientific (Australia); 3,3,5-triiodo-l-thyronine sodium salt (T3), adenine, transferrin, hydrocortisone, insulin, tris(hydroxymethyl)aminomethane, and EDTA from Sigma-Aldrich; and isoproterenol from Merck (Sydney, Australia). Sterile ethanol 70% *v*/*v* for sterilization was supplied by ORION Laboratories (Balcatta, Australia).

Unless specified otherwise, all per cent concentrations or compositions in this report are expressed in percentage by weight.

### Preparation of fibroin and sericin solutions

The solution of regenerated BMSF was prepared according to a protocol we have previously established (Chirila et al. 2008). The concentration of solution used in experiments was 1.78% (by gravimetric analysis).

To prepare the *B. mori* silk sericin (henceforward BMSS) solution, a published protocol (Nagura et al. 2001) was followed with some modifications. Cocoons (6 g) were cut into 1 cm × 1 cm pieces and washed with water in a 5-L beaker with vigorous stirring for 30 min at room temperature. The washed material was transferred in a screw-capped bottle, with 80 mL water, and autoclaved at 121°C for 30 min. The resulting solution was passed through a paper filter to retain the undissolved fibroin material, then aspirated in a syringe and injected into dialysis tubing (MWCO 12.4 kDa) that was pre-treated by soaking in water for 4 h and changing the water five times. The tube was then placed in a 2-L beaker with hot water (80°C) and dialysed with stirring for 4 h and three water exchanges. The final solution was removed from the tube, filtered through the 0.7-μm Minisart^®^ filter, and collected in a glass container that was kept at 80°C (to prevent gelation) until use. The concentration of BMSS in the solutions resulting from various batches was between 1.12% and 1.24% (by gravimetric analysis).

### Preparation of membranes

The BMSF and BMSS membranes were cast from their respective solutions. To produce the blended membranes, the two solutions were mixed before casting at BMSF/BMSS weight ratios of 90/10, 50/50, and 10/90, respectively. For casting, 45-mm glass Petri dishes were first coated with a film of Topas^®^ polymer by the evaporation from a solution in cyclohexane. The solutions were prepared starting with 2 mL of BMSF solution and adding the calculated volumes of the other components. Each solution (BMSF, BMSS, and three mixtures) was poured into the dish, and all dishes were placed in a fan-driven oven and kept for 12 h at room temperature (22°C to 23°C). The dishes with dried membranes were placed in a vacuum enclosure to be annealed in the presence of a container with water, under a vacuum of -80 kPa at room temperature for 24 h. The membranes were then peeled off the Topas^®^ supporting films, and further used in experiments. The thickness of all prepared membranes was approximately 10 μm.

### Crosslinking of the BMSF/BMSS 50/50 blend

The blended membrane with the composition BMSF/BMSS 50/50 was prepared also as a crosslinked material for comparative assessment. The crosslinking was carried out with genipin, employing a slightly modified published procedure (Motta et al. 2011). An amount of genipin equivalent to 12% of the final protein content was mixed with 3 mL solution of 1.78% BMSF and stirred for 5 h at 40°C, when 4.6 mL solution of 1.16% BMSS were added. The reaction mixture acquired a light blue hue, which is the hallmark of the reaction between genipin and amino acids (Djerassi et al. 1960). After additionally stirring for 1 h at 40°C, the product was filtered successively through the 0.7-μm and 0.2-μm Minisart^®^ filters. The resulting solution was processed into a membrane as described above, except for the water-annealing treatment. Following the first drying, the membrane was washed thoroughly in water, dried again, and peeled off the Topas^®^ supporting film.

### Gel electrophoresis analysis of sericin

The molar mass distribution of BMSS was investigated by sodium dodecyl sulphate-polyacrylamide gel electrophoresis (SDS-PAGE), using a Novex^®^ XCell Sure Lock™ Mini-Cell system (Life Technologies Inc, Carlsbad, CA, USA) and an EPS-250 Series II Power Supply unit (CBS Scientific Company Inc, San Diego, CA, USA). The sericin solution was mixed with both NuPAGE^®^ sample preparation reagent and NuPAGE^®^ sample reducing agent, and heated at 70°C for 10 min. A volume of BMSS solution containing approximately 50 μg protein was loaded into a 1-mm thick 3% to 8% NuPAGE^®^ Novex^®^ Tris-acetate gel in NuPAGE^®^ Tris-acetate SDS running buffer. The gels were run at a voltage of 150 V for 1 h together with a HiMark™ Pre-stained Protein Standard (Life Technologies). The resulting gel was washed in three 5-min stages with distilled water and soaked in SimplyBlue™ SafeStain solution (Life Technologies) containing Coomassie^®^ G-250 stain for 1 h under gentle stirring. The gel was washed in distilled water for 1 h and then photographed.

### Mechanical testing of membranes

From each membrane, strips (1 × 3 cm) were cut out and subjected to tensile measurements (for stress, modulus, and elongation) in an Instron 5848 microtester (Instron, Wycombe Buckinghamshire, UK), equipped with a 5 N load cell and a set gauge distance of 14 mm. The samples were loaded by pneumatic grips and submersed in phosphate buffer solution (pre-heated to 37°C ± 3°C) in a BioPuls™ unit for 5 min prior to stretching. Stress–strain plots were recorded, the Young’s moduli were computed in the linear region, and elongation at break was also measured. The mean values were calculated from results generated by six measurements for each specimen, which were cut out from at least three different membranes for the same composition.

### Analysis by Fourier transform infrared-attenuated total reflectance spectroscopy

The Fourier transform infrared-attenuated total reflectance (FTIR-ATR) spectra of the films (fibroin, sericin, and blends) were collected using a Nicolet Nexus 5700 FTIR spectrometer (Thermo Electron Corp, Marietta, OH, USA), equipped with a Nicolet Smart Endurance diamond ATR accessory. Each spectrum was obtained by co-adding 64 scans in the 4,000 to 525 cm^–1^ range at a resolution of 8 cm^–1^. Spectra were recorded and analyzed using OMNIC 7 software package (Thermo Electron Corp).

### Contact angle analysis

Membranes of BMSF, BMSS, and their blends were cast as films and annealed directly onto microscope glass slides, and they were not removed prior to measurements. The genipin-crosslinked 50/50 blended film was not annealed. The contact angles were measured after placing a water droplet onto the dry films. A water droplet of about 5 μL was applied onto each surface, and photographs were taken with a Sanyo VCB-3512 T CCD camera (Sanyo Electric Co, Moriguchi, Osaka, Japan) at an interval of 5 s after the droplet was dispensed. The resulting contact angle was measured in a goniometer (FTÅ200, First Ten Ångstroms Inc, Portsmouth, VA, USA) using the FTÅ Drop Shape Analysis Software Version 2.0 (2002). The results reported are the average values of 16 measurements for each composition.

### Establishment of primary human corneal limbal epithelial cell cultures

The protocol for this stage was detailed in a previous paper (Bray et al. 2013). The ocular tissue was harvested as corneoscleral rims or caps from two different donors, provided by the Queensland Eye Bank (Brisbane, Australia), with donor consent and regulatory ethics approval. In brief, after washing, sectioning, and incubating with 0.25% dispase, the dissociated human limbal epithelial (HLE) sheets were collected, pooled, centrifuged, and re-suspended in 0.25% trypsin in EDTA. After further washing and centrifugation, the HLECs were suspended in serum-supplemented culture medium and propagated in the presence of irradiated 3T3 murine fibroblast feeder cells as described elsewhere (Bray et al. 2011). The passage 1 cultures were further used in the cell attachment assay and in a 3-day cell growth experiment.

### Cell attachment assay

The attachment of cells was evaluated according to the manufacturer’s instructions for the Quant-iT™ PicoGreen^®^ dsDNA assay (Life Technologies), as detailed in a previous report (Bray et al. 2013). In brief, 2 × 10^4^ HLECs/cm^2^ were seeded onto BMSF, BMSS, and their blends, which had been deposited each as coatings onto the bottom of wells of a sterilized 24-well plate. The cells were incubated for 4 h in serum-free medium and washed in PBS. After adding 1 mL of 0.1% Triton-X100, the plates were incubated at room temperature for 1 h. Each well was then triturated and the supernatants were centrifuged. To carry out the PicoGreen analysis, 25 μl of each sample were aliquoted into a 96-well plate with 75 μl of TE buffer. PicoGreen dye was then added at a dilution of 1:200 in the same buffer to each well in 100 μl portions. The plate was read on a fluorescent microplate reader (FLUOstar OPTIMA, BMG Labtech Pty Ltd, Mornington, Victoria, Australia), at the wavelengths 480 nm (excitation) and 520 nm (emission). Cell attachment was assessed based on the DNA content (calculated and plotted in ng/mL), which relates directly to the number of cells.

These experiments were conducted in triplicate for each series of experiments with cells obtained from two different tissue donors. The results were statistically processed by the one-way analysis of variance (ANOVA) in conjunction with Tukey-Kramer multiple comparisons, using the GraphPad Prism^®^ version 6.0. Bright-field microscopy was used to visualize and photograph the 3-day cell cultures in a Nikon TE2000-U (Chiyoda-ku, Tokyo, Japan) instrument.

## Results and discussion

### The primary and secondary structures of sericin

Historically, sericin in *B. mori* silk triggered the scholars’ interest quite early in the development of the modern scientific investigation. The earliest report on sericin was published in 1785 by l'Abbé ('Father’) Collomb (Collomb 1785), who was the first to demonstrate its solubility in hot water. A rigorous study of the effects of water, alcohol, alkaline solutions, soap, and acids, and exposure to light on the silk yarn was published two decades later (Roard 1808), showing that the portion of the silk soluble in aqueous or alcoholic media contained at least four fractions, and two of them were assumed to be constituents of the gum coating, i.e. what we call now sericin. Throughout the next century, research has been focused on improved methods to remove and isolate sericin and to identify its constituents, as overviewed in some earlier publications (Shelton and Johnson 1925; Lucas et al. 1958). The extraction in boiling water or by autoclaving became the techniques of choice for extracting sericin from raw silk or cocoons.

**Figure 1:**
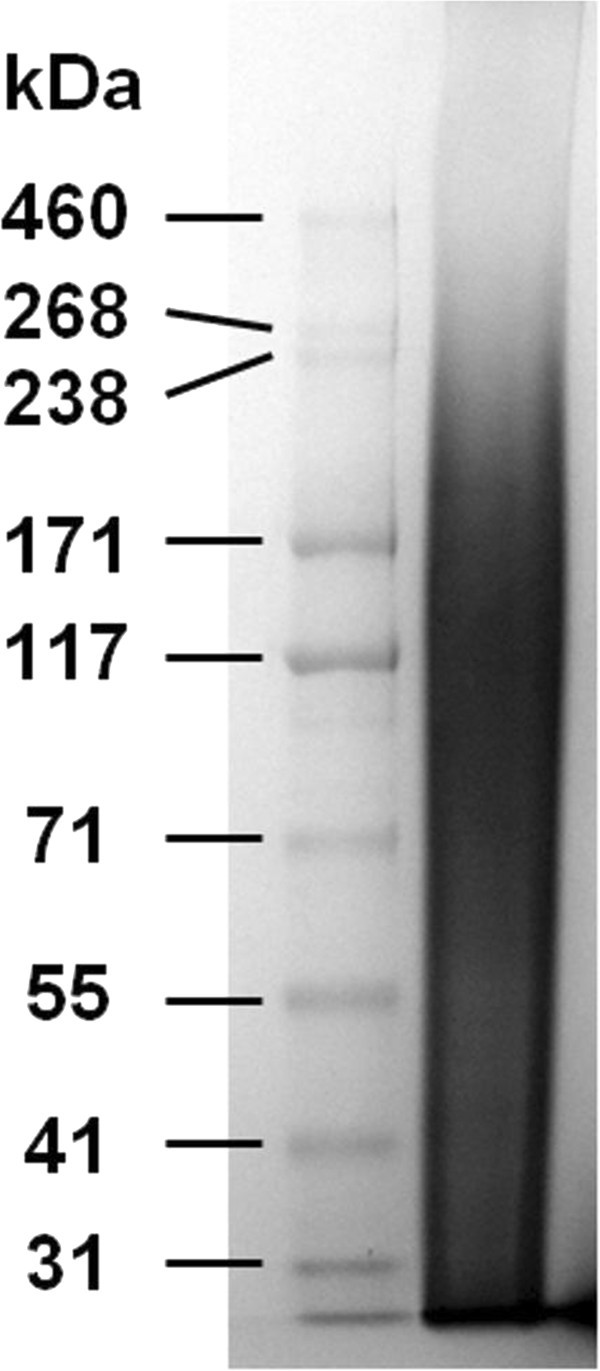
**Analysis of sericin by SDS-PAGE.** Electrophoretic pattern of sericin regenerated from *B. mori* cocoons (right lane). Left lane shows the molar mass marker positions (see text for details of the analysis).

We have investigated by SDS-PAGE the electrophoretic mobility of the sericin components in order to visualize the distribution of their molar masses. Staining revealed a smear pattern (Figure 1), which strongly suggests that the polypeptides in sericin were degraded during processing by autoclaving owing to hydrolytic reactions. Our finding is similar to the smear patterns reported by many investigators for both fibroin and sericin after being processed in aqueous media at elevated temperatures. It is known that the sericin harvested directly from the silkworm’s middle silk gland (MSG) display electrophoretic patterns with distinct protein bands, unlike the sericin processed from cocoons in conditions of high temperature. Confirmation of the effect of autoclaving on sericin has been recently provided (Teramoto et al. 2005,2006) in experiments where pure sericin was collected from the silkworms belonging to a new race known as 'Sericin Hope’ that produces exclusively sericin. This race was developed in Japan through the breeding of mutant silkworms *Nd* or *Nd-s*, which practically synthesize sericin only (Julien et al. 2005), with the strain KCS83, a high cocoon-producing species (Mase et al. 2006). Sericin collected directly from the cocoons (Teramoto et al. 2005) or glands (Teramoto et al. 2006) of the Sericin Hope silkworms exhibited an electrophoretic pattern comprising at least five distinct polypeptides; however, these bands could no longer be identified following autoclaving of the samples as they merged into a smear pattern practically identical to that shown in Figure 1. Indeed, temperature is an important factor in the degradation of sericin proteins: upon isolation of sericin from cocoons at room temperature using ethylenediamine/cupric hydroxide (Tokutake 1980) or lithium thiocyanate (Takasu et al. 2002), distinct polypeptide bands could be identified in the electrophoretograms.

Unlike BMSF, which is known to be composed of three well-defined polypeptidic subunits, with the approximate molar masses of 360, 26, and 30 kDa (Inoue et al. 2000; Julien et al. 2005), the number and molar mass of the subunits in BMSS are still disputed. Long time ago, it was noticed that BMSS is a complex mixture of polypeptides in a number that can be as high as 15 (or even higher) according to some investigators (Sprague 1975). Throughout a century of research, different investigators found various numbers of polypeptide fractions in BMSS, such as 2 (Kondo 1921; Shelton and Johnson 1925; Kodama 1926), 3 (Mosher 1934; Rutherford and Harris 1940; Bryant 1948; Kikkawa 1953; Tashiro and Otsuki 1970; Takasu et al. 2002), 5 (Gamo et al. 1977), 6 (Tokutake 1980), or more (Sprague 1975; Fedič et al. 2002), although it was also proposed (Rutherford and Harris 1940) that the existence of fractions is an artefact caused by degradation and that sericin is a homogeneous material. In terms of molecular mass, the range encompassing all reported values is between 20 and 400 kDa. Genomic analysis of sericin showed that the genes *Ser1*, *Ser2*, *Ser3*, *MSG-3*, *MSG-4*, and *MSG-5* encode for sericin in *B. mori* silkworm’s middle gland (Gamo 1982; Michaille et al. 1986,1989; Grzelak 1995; Julien et al. 2005; Kundu et al. 2008), and therefore at least six major polypeptides are expected to be synthesized in the gland, such providing compelling justification for its heterogeneous composition.

**Figure 2:**
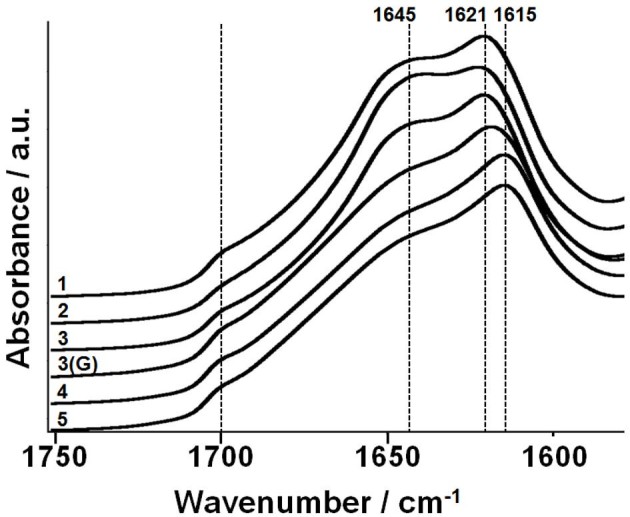
**The Amide I region in the FTIR-ATR spectra of membranes.** The numbers on the recorded spectra represent the membrane compositions (in% by weight): 1, BMSF 100; 2, BMSF/BMSS 90/10; 3, BMSF/BMSS 50/50; 3(G), BMSF/BMSS 50/50/crosslinked with genipin, not annealed; 4, BMSF/BMSS 10/90; 5, BMSS 100.

The FTIR-ATR spectra in the 'Amide I’ region (1,590 to 1,720 cm^–1^) of films of BMSF, BMSS and their blends after water annealing (for 24 h), as well as the BMSF/BMSS 50/50 blend crosslinked with genipin (but not annealed), are all shown in Figure 2. This spectral region is traditionally used for the characterization of the secondary structure of silk proteins. For BMSF, the absorption bands in this region can be assigned as follows: 1,610 to 1,630 and 1,695 to 1,700 cm^–1^ as β-sheet structure; 1,640 to 1,650 cm^–1^ as random-coil structure; 1,650 to 1,660 cm^–1^ as α-helix; and 1,660 to 1,695 cm^–1^ as β turns (Hu et al. 2006,2011). The spectrum of BMSF film (sample 1) showed peaks at 1,621 and 1,700 cm^–1^, indicating that the sample had a significant amount of β-sheet crystal component. For the BMSF/SS 90/10 blend film (sample 2), the peak at 1,621 cm^–1^ was smaller than that of sample 1 in relation to the band at 1,645 cm^–1^ corresponding to the random-coil structure. In the spectrum, we can see a decrease of the β-sheet band that indicates lower crystallinity of sample 2 as compared to sample 1. This is due to the presence of sericin that has been found to retard the crystallization of fibroin (Lee 2004). In the spectra of samples 3 and 4, we can see that while the β-sheet band decreases in intensity, a band at a lower wavenumber (1,615 cm^–1^) increases in intensity, such indicating the predominance of sericin. The spectrum of BMSS film (sample 5) displays an intensive band at 1,615 cm^–1^ as well as a shoulder at 1,700 cm^–1^, both corresponding to β-sheet structures and their aggregates (Teramoto and Miyazawa 2005; Teramoto et al. 2005; Teramoto et al. 2008) and signifying a higher crystallinity of BMSS when compared to that of BMSF. The spectrum of the crosslinked sample 3(G) suggests slightly larger β-sheet content than that in the uncrosslinked sample.

### Tensile properties of membranes

**Figure 3:**
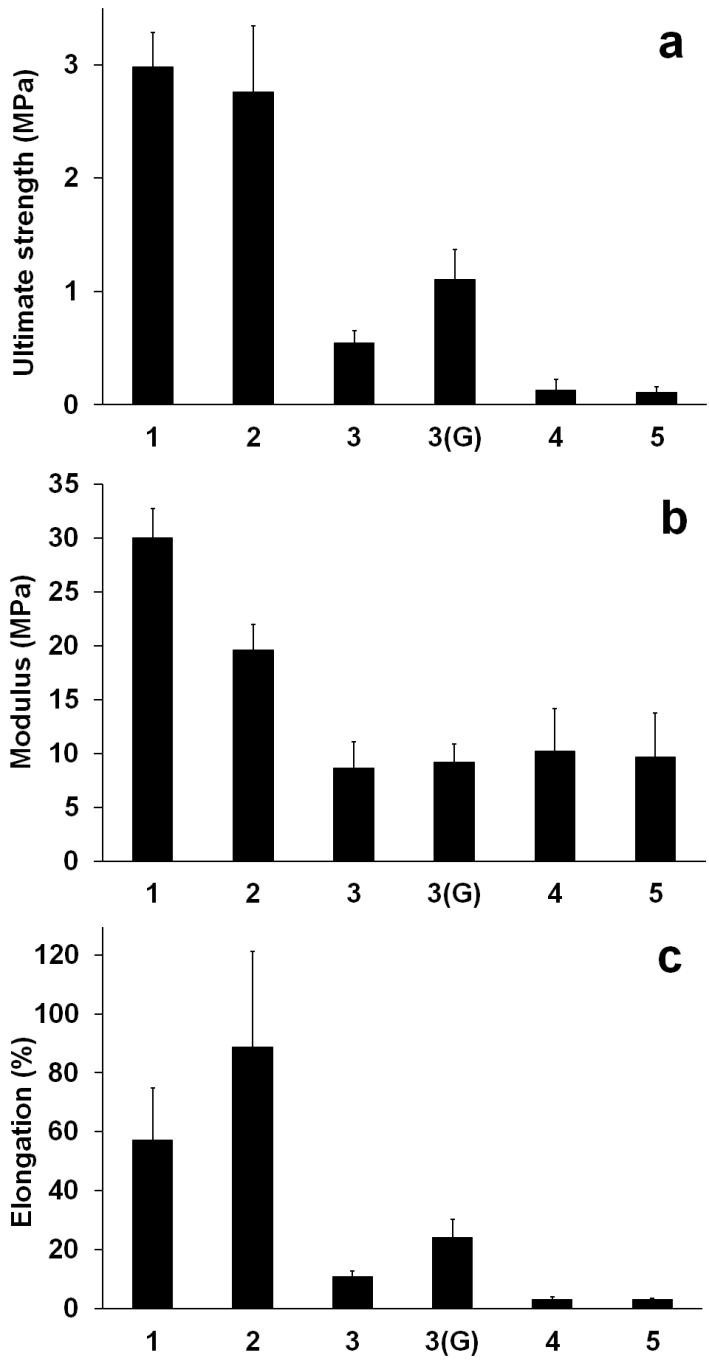
**Quantitative comparison of the tensile characteristics of membranes. (a)** Ultimate tensile strength. **(b)** Young’s modulus. **(c)** Elongation at break. The numbers on the abscissa represent the membrane compositions as indicated in the caption to Figure 2. Except for sample 3(G), all samples were water-annealed prior to measurements.

The results of mechanical testing (Figure 3) indicate poor tensile characteristics for BMSS. Its strength, stiffness (elastic modulus), and elasticity are much lower than those of fibroin. This is expected, as sericin is a globular protein rather than fibrous. It may be also due to the hydrolytic degradation of sericin during processing. For instance, mechanical properties that were significantly superior to those measured in the present study were reported (Teramoto et al. 2008) for the sericin isolated from Sericin Hope cocoons under mild conditions (35°C). On the other hand, sericin extracted by autoclaving could not even be subjected to tensile measurements, as the films were too fragile and broke 'merely by lifting by pincette’ (Nagura et al. 2001).

The higher the content of sericin in the blends, the lower their tensile characteristics. When comparing the blends to fibroin, as a confirmation of our results in Figure 3 (sample 2), it was found (Motta et al. 2011) that a BMSF/BMSS 90/10 blend displayed lower tensile strength and modulus, but a slightly higher elongation at break. Crosslinking with genipin of the BMSF/BMSS 50/50 blend was carried out to assess possible improvement of the mechanical properties. Indeed, it enhanced the strength and elasticity, but not the modulus (Figure 3, sample 3(G)), which basically remained the same.

A slight reduction of stiffness has been reported upon crosslinking sericin with dimethylolurea (Nagura et al. 2001) or upon crosslinking the blend BMSF/BMSS 90/10 with either genipin or poly(ethylene glycol) diglycidyl ether (PEG-DE) (Motta et al. 2011), which has been explained, respectively, either by an effect of the change in the water content of the films (which in its turn depends on the crosslinking degree) or as a consequence of pore formation due to the removal of sericin by alcohol treatment. In our study, however, the membranes were not subjected to alcohol treatment prior to testing. With regards to the water content (WC) of BMSF/BMSS 50/50 samples, we measured 58.51% ± 0.92% for the crosslinked one and 52.57% ± 3.54% for the uncrosslinked water-annealed sample, respectively. However, this difference did not affect the stiffness of the membranes. For comparison, the measured WCs of pure fibroin (sample 1) and of pure sericin (sample 5) were 36.01% ± 1.70% and 53.37% ± 2.53%, respectively.

When compared to the tensile strength of the natural human cornea, reported to be around 3.8 MPa (Zeng et al. 2001), sericin-based membranes cannot compete, and even fibroin is less strong. However, the values of Young’s moduli for BMSF, BMSS, or blends are higher than those reported (Hjortdal 1996) for the human cornea (9 MPa for meridional loading and 13 MPa for circumferential loading). The potential ways to improve the mechanical properties of BMSS include chemical crosslinking, mild processing conditions to prevent degradation, or blending with other macromolecular materials. To achieve these aims, work is in progress in our laboratories.

### Contact angles

Table 1 shows the contact angle values measured for films of BMSF, BMSS, and their blends. The values may reflect in part the vagaries associated with this type of measurement, although we carried out an inordinately large number (*N* = 16) of measurements for each composition. BMSF is clearly more hydrophilic than BMSS, and there is an obvious trend in blends of decreasing hydrophilicity (i.e. higher contact angles) with the increase of BMSS content, although it is difficult to explain why the blends 50/50 and 10/90 displayed contact angles greater than BMSS as such. The crosslinking with genipin induced enhanced hydrophilicity of the film surface.

**Table 1:** **The contact angles of water on the film surfaces**

Sample	BMSF/BMSS (% by weight)	Contact angle (deg)^a^
1	100/0	41.7 ± 0.4
2	90/10	51.3 ± 0.6
3	50/50	53.7 ± 1.1
3(G)^b^	50/50	58.4 ± 0.6
4	10/90	59.0 ± 1.2
5	0/100	53.5 ± 1.4

^a^The results are given as mean values ± SEM for *N* = 16; ^b^Sample was crosslinked but not annealed.

It may be that the measured values are related to the content of β-sheet in the conformation of sericin leading to indirect effects on the hydrophilicity; but irrespective of possible explanations, the important fact is that the values in Table 1 place all compositions in the category of materials that promote cell attachment (Horbett and Klumb 1996).

### Biocompatibility of sericin and its blends

Our rigorous examination of the available literature suggested that blaming exclusively sericin for sensitization to silk was rather speculative and that, in many instances, publications on this topic have been misinterpreted or misquoted. In some early reports on human allergic sensitivity to silk (Clarke and Meyer 1923; Figley and Parkhurst 1933), sericin was indeed suggested as a cause, but only because its solubility in water. Most of the studies published at the same time or later (Taub 1930; Friedman et al. 1957; Brown and Coleman 1957; Kino and Oshima 1979; Häcki et al. 1982; Johansson et al. 1985; Harindranath et al. 1985; Nakazawa and Umegae 1990; Suzuki et al. 1995; Celedón et al. 2001) could not confirm this suggestion. Other investigators also have initially proposed sericin as a pathogen (Zhaoming et al. 1990) only to play down later this assertion (Zhaoming et al. 1996).

There are, however, arguments that appear to support the sensitizing effect of sericin, like in a study (Soong and Kenyon 1984) on the post-operative complications in patients who underwent cataract surgery and were sutured with virgin silk (i.e. containing sericin). However, a previous *in vivo* animal study (Salthouse et al. 1977) had shown that the absence of sericin in degummed sutures did not actually prevent tissue responses. In the reference most frequently cited as a confirmation that sericin is a pathogenic allergen (Dewair et al. 1985), the investigators concluded that the allergenic polypeptides 'are more likely to belong to the sericin group of silk proteins’, a statement that was rather tenuously based on the molecular weights of 12 polypeptides isolated by gel electrophoresis.

In recent papers (Panilaitis et al. 2003; Aramwit et al. 2009a; Hakimi et al. 2010), convincing evidence was provided that sericin does not induce inflammatory or cytotoxic effects. Sericin-mediated activation of a macrophage cell line has been shown to be related *not* to sericin itself but to a physical association between sericin and fibroin, and an inflammatory response could be triggered only in the presence of another pro-inflammatory agent (Panilaitis et al. 2003). Moreover, in experiments involving the growth of human microvascular endothelial cells in the presence of sericin isolated from domesticated or wild silkworm cocoons, no cytotoxic/cytostatic effects have been seen; on the contrary, supplementation with sericin has led to significant enhancement of cell proliferation (Hakimi et al. 2010). These studies alone would exonerate sericin of its potential cytopathologic activities. Moreover, a large number of studies demonstrated the general biocompatibility of sericin and led to many applications involving its contact with body tissues (Kundu et al. 2008; Sehnal 2011).

Considerably fewer reports are available on the use of sericin as a substratum or scaffold for growing cells and tissues. In an early study (Minoura et al. 1995b), BMSS harvested directly from the silk gland was evaluated as a substratum for mouse fibroblasts (line L-929), alone or blended with fibroin. Cell attachment and proliferation on the two silk proteins were similar to those on collagen and higher than on tissue culture plastic. The ability of BMSS, either directly extracted from MSG or regenerated from cocoons, to function as a substratum for cell growth was latter confirmed by others (Terada et al. 2002; Tsubouchi et al. 2005; Xie et al. 2007; Aramwit et al. 2009b,^2010^).

**Figure 4:**
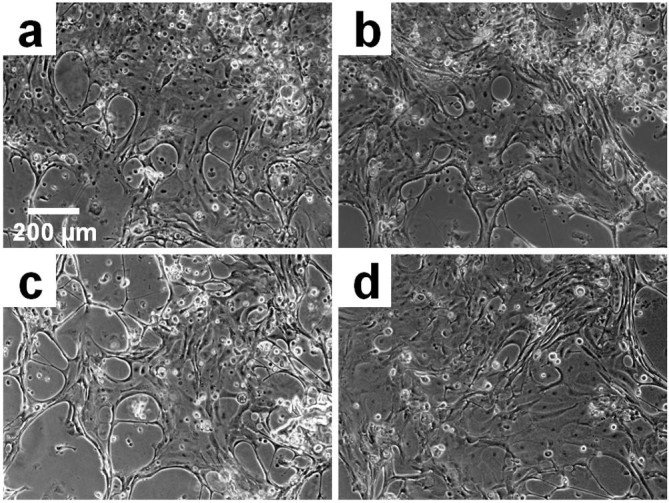
**Growth of primary human corneal limbal epithelial (HLE) cells after 3 days in culture.** The micrographs show the morphology of cells growing on **(a)** tissue culture plastic (control), **(b)** BMSF, **(c)** BMSF/BMSS 10/90 blend, and **(d)** BMSS. The scale bar is the same for all panels.

**Figure 5:**
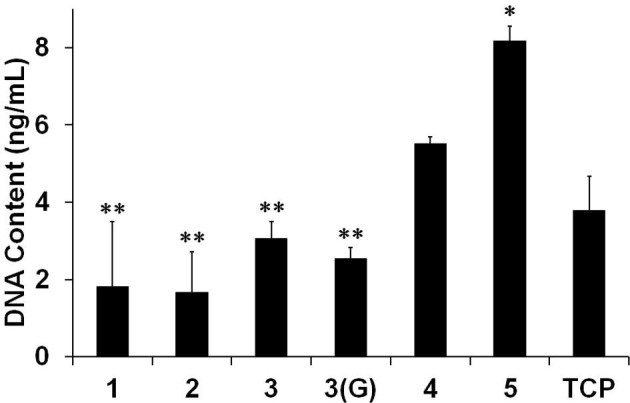
**Bar chart showing a quantitative comparison of the HLE cell attachment to BMSF, BMSS, and their blends.** The cells were seeded in the absence of serum. The bars represent mean values ± SEM for the total number of viable cells after 4 h estimated from the DNA content using the PicoGreen^®^ dsDNA assay (see text for details). The numbers on the abscissa represent the membrane compositions as indicated in the caption to Figure 2. TCP denotes 'tissue culture plastic’ (control sample). The differences between the sample marked with a single asterisk and each of those marked with double asterisk are statistically significant (*p* < 0.05). Except for sample 3(G), all samples were water-annealed prior to use as substrata.

We aim at extending the range of sericin’s potential use as a substratum or scaffold for cells in ophthalmic tissue engineering. As mentioned in the introductory section, research in this field has been limited so far to the BMSF membranes. In the present study, the *in vitro* attachment and growth of HLECs on BMSS membranes was investigated to gauge the cell response to sericin. The primary cell cultures established from donor human eye tissue displayed characteristic cobblestone morphology and reached confluence within 2 weeks or so. After being passaged and then re-suspended in serum-free culture medium in the presence of BMSS membranes, the cells attached to the substratum and maintained their viability and morphology, as seen after 3 days of growth (Figure 4). There is no difference, from one substratum to another, regarding the morphology of cells. However, the level of cell attachment was variable, as shown in Figure 5. A dose-dependent increase with the BMSS content is evident with respect to the level of cell attachment. The level of attachment to pure sericin (sample 5) is higher than that to fibroin (sample 1) or a fibroin-rich blend (sample 2), and even to 50/50 blends (samples 3 and 3(G)). These differences are statistically significant and provide compelling evidence of the suitability of BMSS as a substratum for the growth of HLECs. Although the level of attachment to a sericin-rich blend (sample 4) is also higher than that to samples 1, 2, 3, and 3(G), these differences are not statistically significant. An enhancement of cell attachment to sericin and to BMSF/BMSS 10/90 blend when compared to the attachment to the tissue culture plastic (TCP) control sample is also evident, although the differences were not statistically significant.

In terms of contact angle values, both BMSF and BMSS are expected to promote cell attachment, but the differences between BMSS (sample 5) and the blend samples 2, 3, and 3(G) (Table 1) are too small to justify the differences in cell attachment shown in Figure 5. Nevertheless, the higher hydrophilicity of BMSF (sample 1) may be related to a lower level of attachment. However, the mechanism of cell attachment to BMSF or BMSS appears to be more complex and has not been elucidated so far. As none of these proteins contains any of the identifiable cell-binding peptide motifs, such as the arginine-glycine-aspartic acid (RGD) sequence, the process of cell attachment has been suggested to be non-specific, likely based on electrostatic interactions (Minoura et al. 1995b). In sericin, according to other investigators, a 170-kDa polypeptide fraction encoded by *Ser1* gene may have an active role in the cell attachment process (Tsubouchi et al. 2005). Our analysis (Figure 1) indicated the presence of polypeptide fractions around this molar mass. However, at this stage it can be only surmised that the cell-adhesive properties of BMSF or BMSS arise from a favourable combination of non-specific interactions promoted by certain characteristics of the substratum’s surface such as charge, wettability, and topography. Further investigations are needed to explain the enhanced cell attachment on BMSS as compared to BMSF.

## Conclusions

The *B. mori* sericin-based materials are not as strong as those based on *B. mori* fibroin, and they may need improvement in this respect for potential use as surgical implants. However, the attachment of human corneal limbal epithelial cells to sericin or sericin-rich blends is superior to that to fibroin or fibroin-rich blends, with no evidence of any cytopathologic response. These findings would strongly recommend sericin as an appropriate material for making substrata or scaffolds for cellular invasion and tissue formation in applications pertaining to the restoration of damaged ocular surface.

## Authors’ original submitted files for images

Below are the links to the authors’ original submitted files for images. Authors’ original file for figure 1 Authors’ original file for figure 2 Authors’ original file for figure 3 Authors’ original file for figure 4 Authors’ original file for figure 5

## Abbreviations

BMSF: *Bombyx mori* silk fibroin
BMSS: *Bombyx mori* silk sericin
EDTA: Ethylenediaminetetraacetic acid
FBS: Foetal bovine serum
FTIR-ATR: Fourier transform infrared attenuated total reflectance
HLEC: Human corneal limbal epithelial cell
MSG: Middle silk gland
MWCO: Molecular weight cut-off
PEG-DE: Poly(ethylene glycol) diglycidyl ether
SEM: Standard error of the mean
SDS-PAGE: Sodium dodecyl sulphate-polyacrylamide gel electrophoresis
TCP: Tissue culture plastic
WC: Water content.
