# Supplementary figures and images for: Evaluation of silk sericin as a biomaterial: in vitro growth of human corneal limbal epithelial cells on Bombyx mori sericin membranes
Source: Prog Biomater. 2013 Nov 28;2:14. doi: 10.1186/2194-0517-2-14 (PMC5151120; doi:10.1186/2194-0517-2-14)

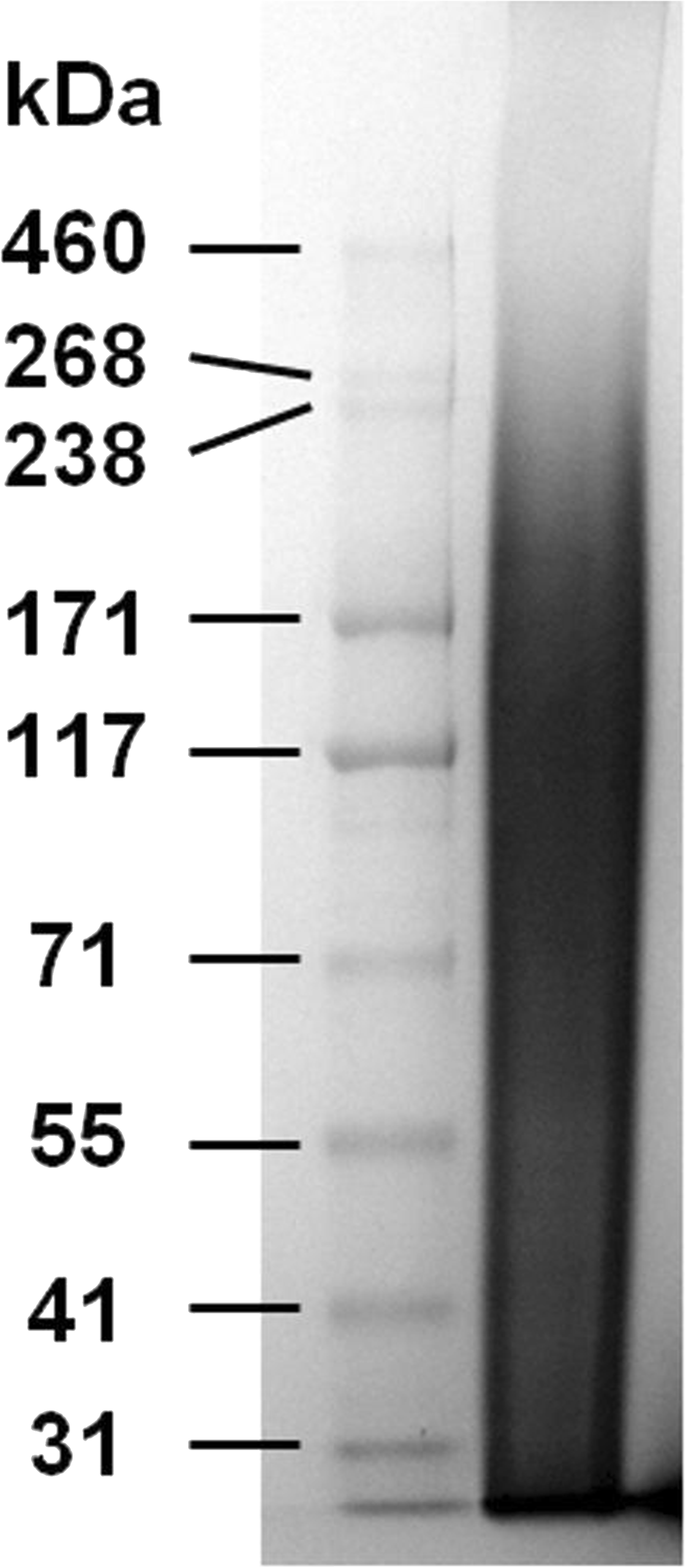

Supplement: Supplementary file 1 — Authors’ original file for figure 1 [file 40204_2013_18_MOESM1_ESM.tiff]

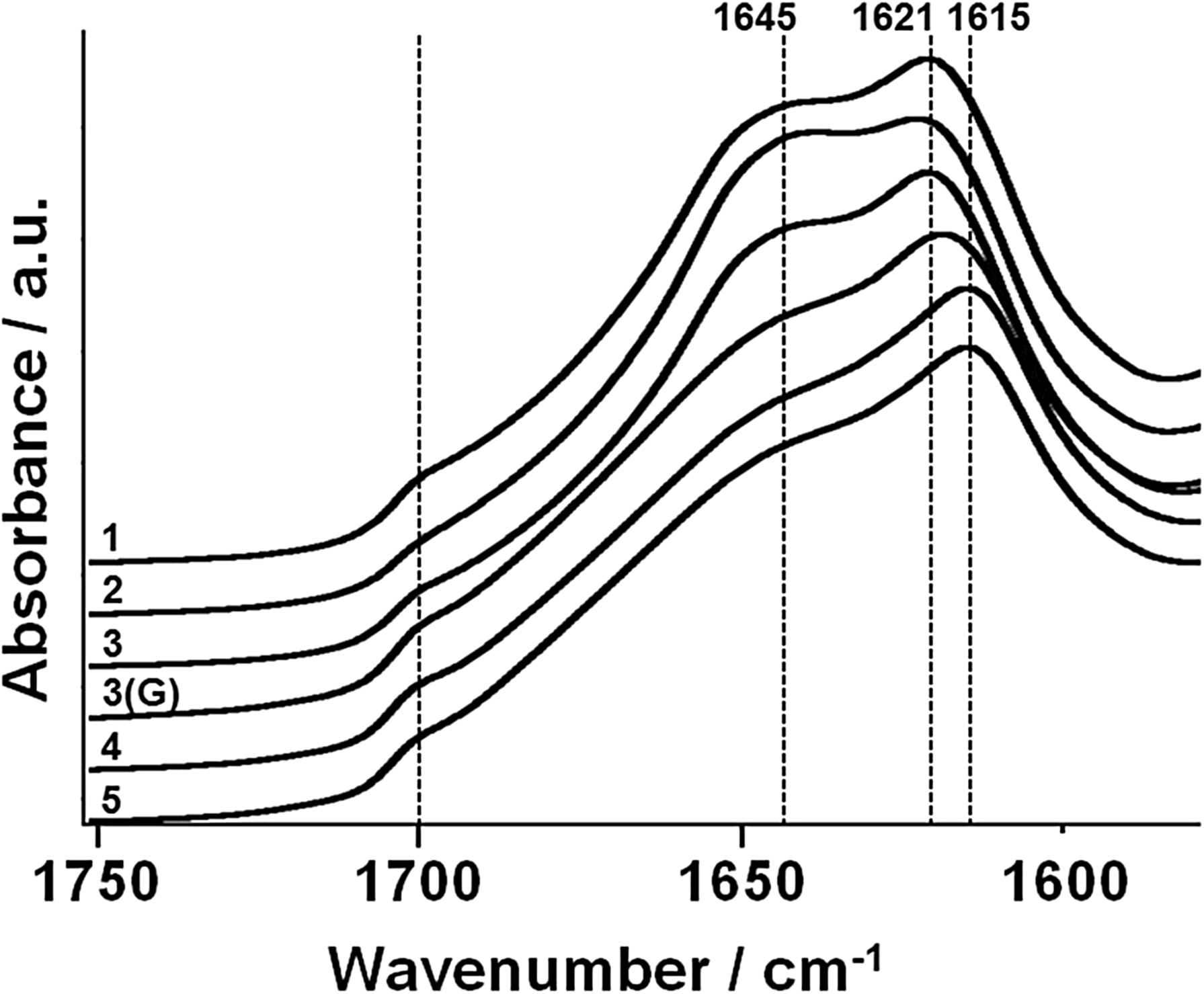

Supplement: Supplementary file 2 — Authors’ original file for figure 2 [file 40204_2013_18_MOESM2_ESM.tiff]

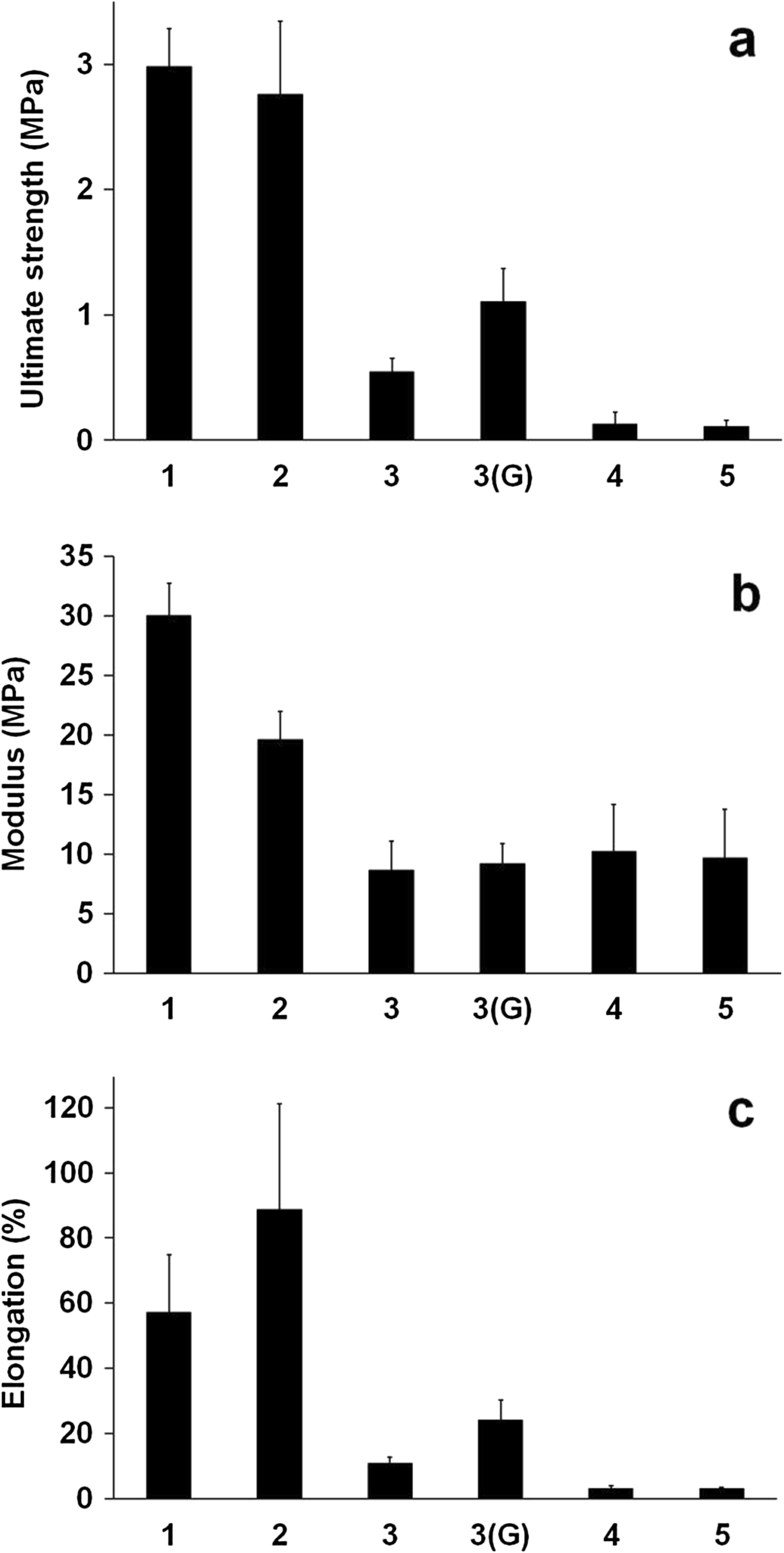

Supplement: Supplementary file 3 — Authors’ original file for figure 3 [file 40204_2013_18_MOESM3_ESM.tiff]

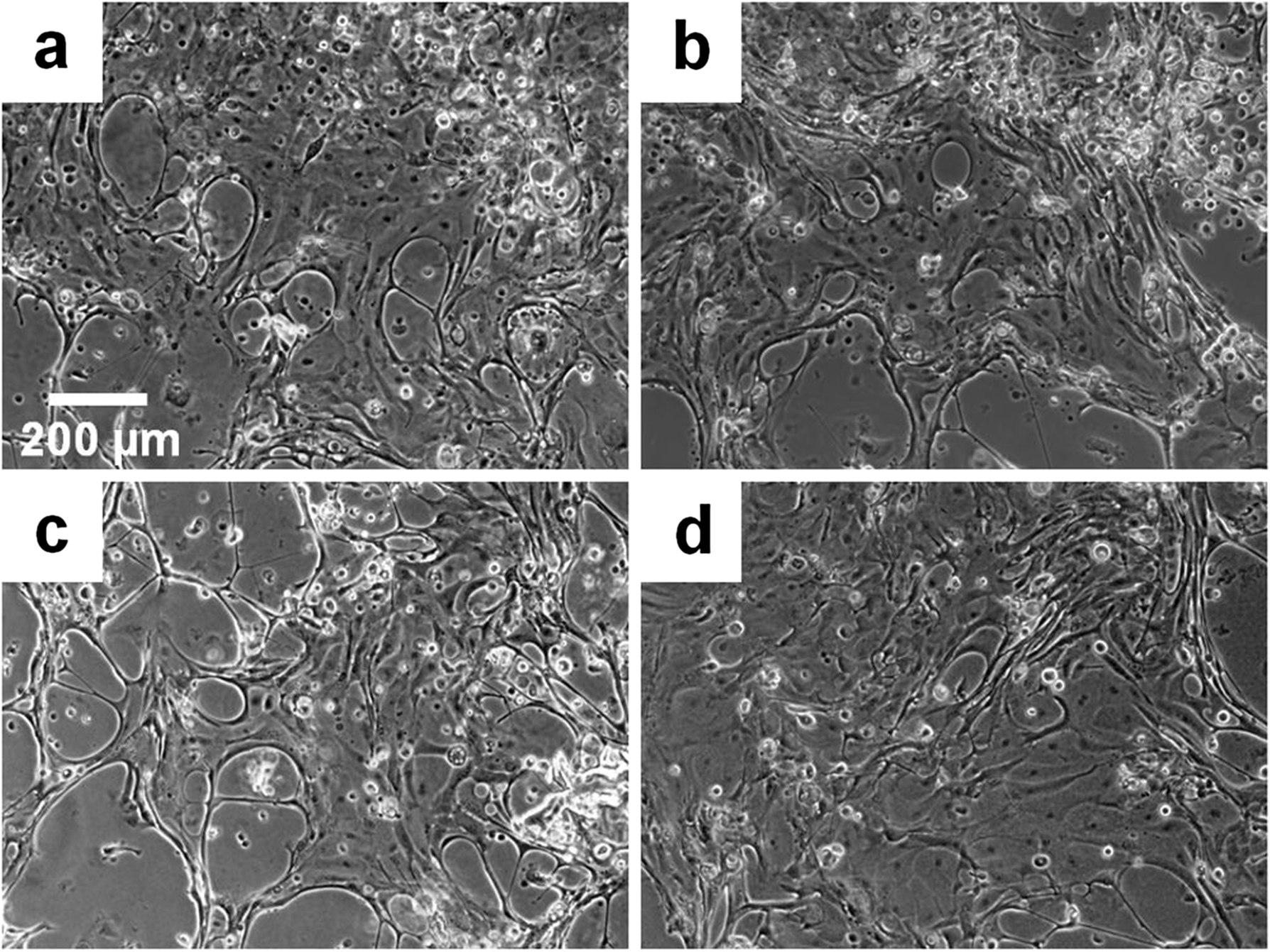

Supplement: Supplementary file 4 — Authors’ original file for figure 4 [file 40204_2013_18_MOESM4_ESM.tiff]

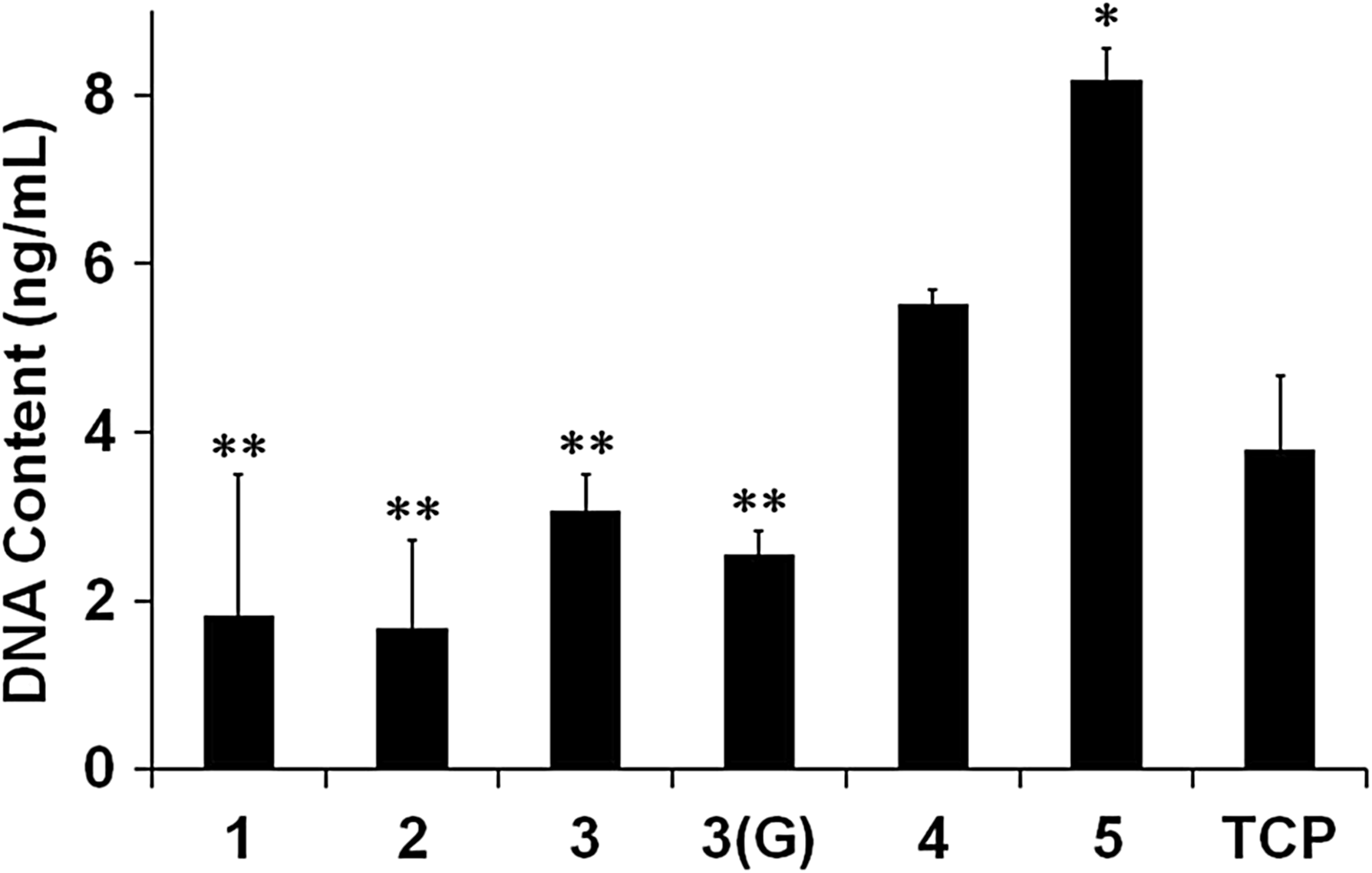

Supplement: Supplementary file 5 — Authors’ original file for figure 5 [file 40204_2013_18_MOESM5_ESM.tiff]
